# Supplementary material for: Machine Learning for Prediction of Immunotherapy Efficacy in Non-Small Cell Lung Cancer from Simple Clinical and Biological Data
Source: Cancers (Basel). 2021 Dec 9;13(24):6210. doi: 10.3390/cancers13246210 (PMC8699503; doi:10.3390/cancers13246210)

Supplementary figures and tables

Table S1: Blood counts at ICI treatment start

Figure S1: Progression-free and overall survival

Table S2: Cox regression: progression-free survival

Table S3: Cox regression: overall survival

Figure S2: Learning curves

Table S1: Blood counts at ICI treatment start

Variable	Mean	Median	Min	Max
Basophils	0.04	0.03	0.00	0.80
Derived NLR	3.15	2.37	0.41	18.58
Eosinophils	0.16	0.11	0.00	3.26
Hemoglobin	11.32	11.30	7.30	16.40
Leucocytes	8.56	7.99	2.09	49.65
Lymphocytes	1.55	1.35	0.11	19.31
Monocytes	0.63	0.58	0.07	1.96
Neutrophils	6.17	5.52	0.86	46.00
NLR	5.66	3.85	0.46	96.91
Platelets	317	290	57	2,013
PLR	273	214	3	2,309

Figure S1: Progression-free and overall survival

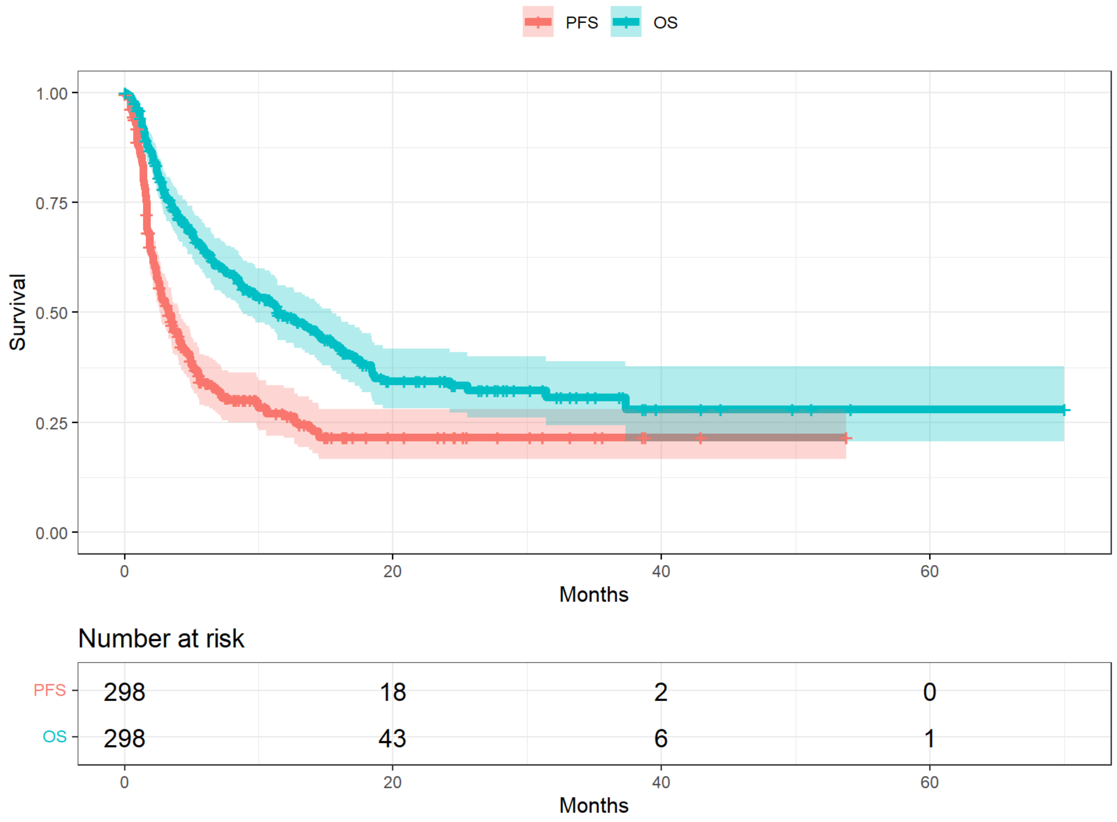

Table S2: Cox regression: progression-free survival

	Univariable cox regression			Multivariable cox regression		
Variable	Hazard ratio [95% IC]	p	signif	Hazard ratio [95% IC]	p	signif
Lymphocytes	0.85 [0.71, 1]	0.0912	.	1 [0.38, 2.6]	0.996	
NLR	1 [1, 1]	0.00585	**	0.71 [0.33, 1.5]	0.376	
Platelets	1 [1, 1]	0.37		0.81 [0.59, 1.1]	0.206	
PLR	1 [1, 1]	0.881		1 [0.66, 1.5]	0.994	
Leukocytes	1.1 [1, 1.1]	0.00246	**	0.69 [0.044, 11]	0.795	
Hemoglobin	0.9 [0.83, 0.97]	0.00525	**	0.84 [0.72, 0.98]	0.0291	*
dNLR	1.1 [1, 1.1]	0.0017	**	1.2 [0.69, 1.9]	0.596	
Neutrophils	1.1 [1, 1.1]	6.26e-05	***	2 [0.15, 26]	0.603	
Monocytes	1.1 [0.66, 1.8]	0.774		1 [0.78, 1.3]	0.885	
Eosinophils	0.53 [0.23, 1.2]	0.138		0.96 [0.77, 1.2]	0.695	
Basophils	0.91 [0.14, 6.1]	0.923		0.99 [0.87, 1.1]	0.917	
BMI	0.96 [0.93, 0.99]	0.0133	*	0.88 [0.76, 1]	0.088	.
Performance status	2.1 [1.6, 2.9]	8.15e-07	***	1.3 [1.1, 1.5]	0.000335	***

Table S3: Cox regression: overall survival

	Univariable cox regression			Multivariable cox regression		
Variable	Hazard ratio [95% IC]	p	signif	Hazard ratio [95% IC]	p	signif
Lymphocytes	0.8 [0.64, 0.99]	0.045	*	1.1 [0.4, 3.1]	0.845	
NLR	1 [1, 1.1]	4.54e-08	***	0.87 [0.43, 1.7]	0.686	
Platelets	1 [1, 1]	0.81		0.83 [0.58, 1.2]	0.305	
PLR	1 [1, 1]	0.331		1 [0.65, 1.6]	0.949	
Leukocytes	1.1 [1, 1.1]	6.63e-07	***	0.99 [0.054, 18]	0.994	
Hemoglobin	0.83 [0.76, 0.91]	3.58e-05	***	0.76 [0.64, 0.92]	0.00392	**
dNLR	1.2 [1.1, 1.2]	8.93e-09	***	1.4 [0.83, 2.2]	0.225	
Neutrophils	1.1 [1.1, 1.1]	4.05e-10	***	1.3 [0.087, 19]	0.857	
Monocytes	1.4 [0.85, 2.3]	0.195		1.1 [0.82, 1.5]	0.558	
Eosinophils	0.13 [0.032, 0.54]	0.00509	**	0.78 [0.55, 1.1]	0.166	
Basophils	0.72 [0.071, 7.4]	0.784		0.99 [0.84, 1.2]	0.943	
BMI	0.97 [0.93, 1]	0.0715	.	0.93 [0.8, 1.1]	0.405	
Performance status	2.8 [2, 3.9]	1.12e-09	***	1.4 [1.2, 1.6]	3.68e-05	***

Figure S2: Learning curves

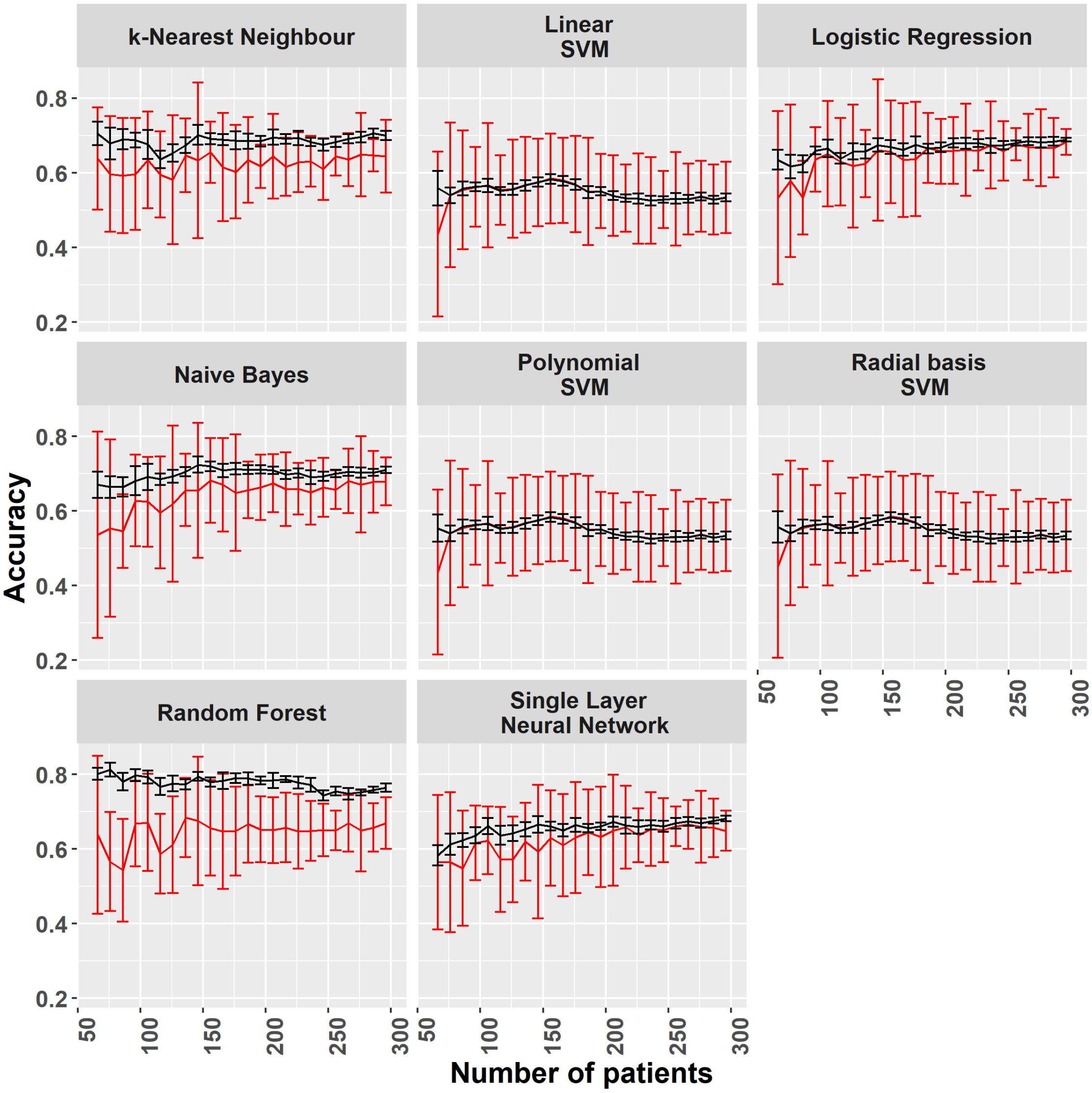

Supplement: Supplementary file 1 [file cancers-13-06210-s001.zip › cancers-1477063-supplementary.pdf]
